# Long-Term Outcomes of Successful Recanalization Compared With Optimal Medical Therapy for Coronary Chronic Total Occlusions in Patients With and Without Left Ventricular Systolic Dysfunction

**DOI:** 10.3389/fcvm.2021.654730

**Published:** 2021-04-20

**Authors:** Lei Guo, Shaoke Meng, Haichen Lv, Lei Zhong, Jian Wu, Huaiyu Ding, Jiaying Xu, Xiaoyan Zhang, Rongchong Huang

**Affiliations:** ^1^Department of Cardiology, The First Affiliated Hospital of Dalian Medical University, Dalian, China; ^2^Department of Radiology, Fuyang Hospital of Anhui Medical University, Fuyang, China; ^3^Department of Cardiology, Beijing Friendship Hospital, Capital Medical University, Beijing, China

**Keywords:** chronic total occlusions, percutaneous coronary intervention, optimal medical therapy, left ventricular systolic dysfunction, LVEF, outcomes

## Abstract

**Background:** The number of coronary chronic total occlusion (CTO) patients with left ventricular (LV) systolic dysfunction is significant, but the clinical outcomes of these patients are rarely reported. The present retrospective cohort study aimed to investigate the long-term outcomes of successful recanalization vs. optimal medical therapy (MT) for CTOs in patients with preserved and impaired LV systolic function.

**Methods:** A total of 1,895 patients with CTOs were stratified according to LV function. Of these, 1,420 patients (74.9%) with LV ejection fraction (LVEF) >45% and 475 patients (25.1%) with LVEF ≤45% were treated with optimal MT or successful CTO percutaneous coronary intervention (PCI). A 1:1 propensity score matching (PSM) was conducted to reduce the impact of potential confounding on the outcomes. The primary outcome was the frequency of major adverse cardiac events (MACEs).

**Results:** Throughout a 2.6-year follow-up and after adjusting for confounders, among patients with preserved LV function, successful CTO PCI was associated with reduced incidence of MACE (14.2 vs. 23.9%, adjusted HR 0.63, 95% CI 0.48–0.83, *p* = 0.001) compared to MT. There was no significant difference in MACE occurrence (29.6 vs. 28.9%, adjusted HR 1.05, 95% CI: 0.71–1.56, *p* = 0.792) between successful recanalization and MT in patients with LV systolic dysfunction. The primary outcome among patients with impaired and preserved LV systolic function after PSM was similar to that from earlier findings before PSM was conducted. A significant interaction between LV function and therapeutic strategy for MACE was observed (interaction *p* = 0.038).

**Conclusions:** Compared to MT alone for management of patients with CTOs, successful CTO PCI may reduce the risk of MACE in patients with preserved LV systolic function, but not in patients with LV dysfunction.

## Introduction

Coronary chronic total occlusions (CTOs) are observed in 10–15% of all patients undergoing coronary angiography and remain one of the most challenging obstacles in coronary intervention (1, 2). The presence of a CTO was found to be the strongest independent predictor of incomplete revascularization in patients with complex coronary artery disease (CAD) undergoing percutaneous coronary interventions (PCIs). It was also associated with higher rates of 4-year mortality and major adverse cardiac and cerebrovascular events (MACCEs) in the Synergy between Percutaneous Coronary Intervention with Taxus and Cardiac Surgery (SYNTAX) trial (3). Some observational studies have reported that CTO-PCI is associated with angina symptom relief, and that it improves long-term survival and left ventricular ejection fraction (LVEF) (4–7). However, CTO-PCI is performed infrequently, due to lesional complexity, procedural complications, and controversial findings, and nearly half of CTO patients are managed by conservative medical therapy (MT) (1, 8–11).

The number of patients with LV systolic dysfunction is significant. Previous studies have reported that approximately 40–53% of patients with CTOs had LV systolic dysfunction (9, 12). However, the clinical outcomes of impaired and preserved LV systolic function in CTO patients have been rarely reported, and there are no current guidelines or consensus related to the optimal treatment strategy for CTO lesions in patients with LV systolic dysfunction. Accordingly, we aimed to assess the long-term outcomes of optimal MT vs. successful CTO recanalization in CTO patients with impaired and preserved LV systolic function.

## Materials and Methods

### Study Design and Population

A total of 27,231 coronary angiography procedures were performed at our center in the period between January 2007 and December 2018. Patients with a confirmed CTO diagnosis were selected. Exclusion criteria were as follows: history of coronary artery bypass grafting (CABG), because CTO lesions in patients with history of CABG surgery show more extensive calcification, negative remodeling, and accelerated progression of atherosclerosis, which are associated with lower success rates, higher rates of complications, and worse outcomes (13); CABG or failed CTO-PCI; acute myocardial infarction (MI); cardiogenic shock; and cancer. A total of 1,895 patients were enrolled in the study. Patients were assigned to revascularization or optimal MT groups according to the initial treatment strategy. Decisions for patients referred for revascularization were based on at least one of the following conditions: (1) presence of symptomatic angina, (2) inducible myocardial ischemia evaluated by echocardiography or myocardial perfusion scan, and (3) myocardial viability on cardiac magnetic resonance imaging (14). MT was strongly preferred in asymptomatic patients who did not have available viability data or in subjects with proven absence of viability. PCI was preferred in symptomatic patients even without information on viability or in asymptomatic patients with viability. The decision to perform PCI for CTO patients was also dependent on several factors, including co-morbidity, the extent of other coronary artery disease, CTO location, technical difficulty, and doctors' and patients' preference (14, 15). Patients' LV systolic function was evaluated using a two-dimensional echocardiogram. Baseline characteristics, echocardiographic data, medications, and procedural data were collected upon study enrollment. Follow-up data were obtained by reviewing medical records and performing telephone interviews with the patients. The study was approved by local institutional review boards in accordance with the principles of the Declaration of Helsinki.

### Definitions and Clinical Endpoints

A CTO is defined as a native coronary artery occlusion with typical appearance (Thrombolysis in Myocardial Infarction (TIMI) grade 0 flow through the lesion with no thrombus, no staining at the proximal cap, and presence of mature collaterals) and definitive corroborating evidence of occlusion duration ≥3 months according to the CTO Academic Research Consortium (CTO-ARC) Consensus Recommendations (16). CTOs in the present study were identified based on medical history or previous coronary angiography. LVEF >45% was defined as preserved LV systolic function, and LVEF ≤45% was defined as LV systolic dysfunction. Successful CTO-PCI was defined as residual stenosis of <20% and restoration of TIMI grade 3 flow after drug-eluting stent implantation. The primary endpoint was the frequency of MACE at follow up. Other endpoints included cardiac death, MI, and target vessel revascularization (TVR). MACEs included cardiac death, MI, and need for TVR. Cardiac death included sudden cardiac death and death due to MI or heart failure. MI was defined as creatine kinase-MB enzyme elevation three times the upper limit of the normal value with ischemic symptoms or electrocardiographic expression of ischemia. TVR was defined as any attempted PCI or surgical revascularization of the CTO target vessel (15, 17). Perioperative MI was defined as a cTn elevation >5 times the 99th percentile of the upper reference limit within 48 h after the procedure in patients with normal baseline values plus the presence of additional supportive electrocardiographic, angiographic, or imaging evidence of ischemia (16).

### PCI Procedures and Medical Treatment

All patients were pre-treated with aspirin and clopidogrel before the procedure. The angiographic characteristics of the CTO lesions were evaluated by dedicated CTO operators before the procedure. Contemporary techniques, such as bilateral injections, microcatheters, novel guidewires, and retrograde approaches, were used in the PCI procedure. The crossing equipment and procedure techniques were used according to the operator's discretion. During the procedure, patients received intravenous unfractionated heparin (100 IU/kg), and activated clotting time (ACT) was repeatedly checked to maintain an ACT of ≥300. Glycoprotein IIb or IIIa inhibitor was administered depending on the operator's choice. After PCI, all patients received 100 mg of aspirin per day indefinitely and 75 mg of clopidogrel per day for at least 12 months. MT included antiplatelet medication, statins, renin-angiotensin system blockade, β-blockers, and nitrate (18). Optimal MT was defined as the use of at least two or more antianginal classes of therapies according to the appropriate use criteria. The medication dosages were maximized as allowed by heart rate, blood pressure, and side effects in the absence of justifiable relative contraindications. The MT was recorded at discharge after the index hospitalization.

### Statistical Analysis

Continuous variables are presented as the mean ± SD and were evaluated using Student's *t*-test. Categorical variables were presented as frequencies and percentage and were compared using the chi-square test. Kaplan-Meier survival curves were plotted to assess long-term outcomes during the follow-up, and the log-rank test was used for comparison between groups. Cox proportional hazards model was used to adjust the potential influence factors. All univariate variables with *p* < 0.1 or clinically relevant variables were included in a statistical model to adjust the hazard ratio (HR). Univariate analysis was performed and the variables included age, sex, smoking, hypertension, diabetes, dyslipidemia, familial history of CAD, history of MI, chronic kidney disease (CKD), baseline medication use, number of CTO lesions, CTO location, multivessel disease, proximal or mid CTO, CTO length, calcification Japanese-chronic total occlusion (J-CTO) score, and SYNTAX score. To reduce the impact of potential confounding on MACEs of the observational study, a 1:1 propensity score matching (PSM) was conducted using all available variables measured based on a multivariable logistic regression to choose patients with comparable baseline data. The nearest-neighbor matching algorithm was used for PSM. Two-sided *p* < 0.05 were considered statistically significant and all analyses were processed using Stata Version 15.1 (StataCorp LLC, TX, USA).

## Results

### Patient Characteristics

Of the 1,895 patients enrolled in this study, 1,420 patients (74.9%) had LVEF >45% and 475 patients (25.1%) had LV systolic dysfunction. A total of 66 patients underwent repeated CTO-PCI procedures, and 45 patients received a successful CTO-PCI. Subsequently, 476 patients underwent a failed PCI (360 patients were with LVEF >45% and 116 patients were with LV systolic dysfunction). Tables 1, 2 show the baseline information for the study patients. Patients with LV systolic dysfunction tended to be male and smokers and had a higher prevalence of previous MI and CKD compared to patients with preserved LV systolic function. The use of β-blocker and angiotensin-converting enzyme inhibitor (ACEI) or angiotensin-receptor blocker (ARB) was more common among patients with LV systolic dysfunction. Angiographic findings revealed that patients in the LV systolic dysfunction group had a higher prevalence of two CTO lesions, multivessel disease, occlusive length ≥20 mm, and higher J-CTO score. No significant differences were found in the prevalence of procedural complications and in-hospital death.

**Table 1:** Baseline clinical characteristics of all patients with and without left ventricular systolic dysfunction, and of patients with and without left ventricular systolic dysfunction stratified according to management.

**Variables**	**Total population**			**Patients with LVEF** **>45%**			**Patients with LVEF** **≤45%**		
	**LVEF >45%**	**LVEF ≤45%**	***P*-value**	**MT**	**Successful PCI**	***P*-value**	**MT**	**Successful PCI**	***P*-value**
	**(*n* = 1,420)**	**(*n* = 475)**		**(*n* = 863)**	**(*n* = 557)**		**(*n* = 350)**	**(*n* = 125)**	
Age, years	64.5 ± 10.1	64.1 ± 10.9	0.328	65.3 ± 10.4	63.4 ± 9.6	0.001	64.0 ± 11.2	64.2 ± 9.8	0.394
Male	1,070 (75.4)	389 (81.9)	0.003	656 (76.0)	414 (74.3)	0.471	288 (82.3)	101 (80.8)	0.711
Smoking	570 (40.1)	238 (50.1)	<0.001	349 (40.4)	221 (39.7)	0.774	171 (48.9)	67 (53.6)	0.363
Hypertension	988 (69.6)	304 (64.0)	0.024	614 (71.1)	374 (67.1)	0.110	225 (64.3)	79 (63.2)	0.828
Diabetes mellitus	526 (37.0)	193 (406)	0.119	342 (39.6)	184 (33.0)	0.012	139 (39.7)	54 (43.2)	0.496
Dyslipidemia	1,060 (75.6)	333 (71.0)	0.120	654 (75.8)	406 (72.9)	0.371	242 (69.1)	91 (72.8)	0.495
Familial history of CAD	165 (11.6)	51 (10.7)	0.600	98 (11.4)	67 (12.0)	0.669	39 (11.1)	12 (9.6)	0.632
Previous MI	393 (27.7)	258 (54.3)	<0.001	263 (30.5)	130 (23.3)	0.003	196 (56.0)	62 (49.6)	0.218
CKD	111 (7.9)	79 (16.9)	<0.001	75 (8.7)	36 (6.5)	0.120	65 (18.6)	14 (11.2)	0.058
Baseline medication									
Aspirin	1,371 (96.5)	456 (96.0)	0.577	829 (96.1)	542 (97.3)	0.209	334 (95.4)	122 (97.6)	0.288
Clopidogrel	1,335 (94.0)	439 (92.4)	0.219	793 (91.9)	542 (97.3)	<0.001	322 (92.0)	117 (93.6)	0.562
Statin	1,357 (95.6)	459 (96.6)	0.313	827 (95.8)	530 (95.2)	0.546	337 (96.3)	122 (97.6)	0.484
β blocker	1,052 (74.1)	386 (81.3)	0.002	637 (73.8)	415 (74.5)	0.771	279 (79.7)	107 (85.6)	0.148
ACEI or ARB	882 (62.1)	332 (69.9)	0.002	550 (63.7)	332 (59.6)	0.118	246 (70.3)	86 (68.8)	0.756

*Values are presented as the mean ± standard deviation or n (%)*.

*ACEI, angiotensin-converting enzyme inhibitor; ARB, angiotensin-receptor blocker; CAD, coronary artery disease; CKD, chronic kidney disease; LVEF, left ventricular ejection fraction; MI, myocardial infarction; MT, medical therapy; PCI, percutaneous coronary intervention*.

**Table 2:** Baseline angiographic, procedural characteristics, and in-hospital outcome of all patients with and without left ventricular systolic dysfunction, and of patients with and without left ventricular systolic dysfunction stratified according to management.

**Variables**	**Total population**			**Patients with LVEF** **>45%**			**Patients with LVEF** **≤45%**		
	**LVEF >45%**	**LVEF ≤45%**	***P*-value**	**MT**	**Successful PCI**	***P*-value**	**MT**	**Successful PCI**	***P*-value**
	**(*n* = 1,420)**	**(*n* = 475)**		**(*n* = 863)**	**(*n* = 557)**		**(*n* = 350)**	**(*n* = 125)**	
One CTO lesion	1,262 (88.9)	377 (79.4)	<0.001	769 (89.1)	493 (88.5)	0.726	286 (81.7)	91 (72.8)	0.035
Two CTO lesions	148 (10.4)	89 (18.7)	<0.001	88 (10.2)	60 (10.8)	0.729	58 (16.6)	31 (24.8)	0.043
LAD	478 (33.7)	174 (36.6)	0.238	263 (30.5)	215 (38.6)	0.002	117 (33.4)	57 (45.6)	0.015
LCX	414 (29.2)	148 (31.2)	0.408	286 (33.1)	128 (23.0)	<0.001	114 (32.6)	34 (27.2)	0.266
RCA	671 (47.3)	245 (51.6)	0.102	407 (47.2)	264 (47.4)	0.931	183 (52.3)	62 (49.6)	0.606
Multivessel disease	1,109 (78.2)	407 (85.7)	<0.001	737 (85.4)	372 (66.8)	<0.001	311 (88.9)	96 (76.8)	0.001
Proximal or mid CTO	993 (69.9)	352 (74.1)	0.083	586 (67.9)	407 (73.1)	0.038	253 (72.3)	99 (79.2)	0.130
Calcification	249 (17.5)	100 (21.1)	0.087	177 (20.5)	72 (12.9)	<0.001	81 (23.1)	19 (15.2)	0.062
length ≥20 mm	887 (62.5)	322 (67.8)	0.037	536 (62.1)	351 (63.0)	0.730	236 (67.4)	86 (68.8)	0.778
J-CTO score	1.60 ± 1.15	1.84 ± 1.18	0.004	1.73 ± 1.23	1.41 ± 1.00	<0.001	1.94 ± 1.21	1.57 ± 1.02	0.001
SYNTAX score	21.7 ± 8.1	23.3 ± 9.0	0.172	22.7 ± 8.6	19.7 ± 7.2	0.006	24.3 ± 9.3	19.3 ± 6.5	0.068
Number of stents	–	–	–	–	1.76 ± 0.95	–	–	1.75 ± 0.91	–
Total stent length, mm	–	–	–	–	29.3 ± 25.3	–	–	31.1 ± 25.4	–
Contrast volume, ml	181 ± 84	166 ± 74	<0.001	–	228 ± 82	–	–	221 ± 66	–
Coronary dissection	18 (3.2)	5 (4.0)	0.711	–	18 (3.2)	–	–	5 (4.0)	–
Coronary perforation	6 (1.0)	2 (1.6)	0.997	–	6 (1.0)	–	–	2 (1.6)	–
In-hospital death	4 (0.3)	4 (0.8)	0.228	–	–	–	–	–	–

*Values are presented as the mean ± standard deviation or n (%)*.

*CTO, chronic total occlusion; J-CTO, Japanese-chronic total occlusion; LAD, left ascending coronary artery; LCX, left circumflex coronary artery; LVEF, left ventricular ejection fraction; MT, medical therapy; PCI, percutaneous coronary intervention; RCA, right coronary artery*.

Among the patients with preserved LV systolic function, 863 patients received MT and 557 patients underwent a successful CTO procedure. Compared to patients in the successful recanalization group, those with MT were older and had more cases of diabetes mellitus prior to MI. In addition, they had more involvement of the LCX coronary artery and complex lesions (multivessel disease, lesion calcification, and high J-CTO and SYNTAX scores).

Among the patients with LV systolic dysfunction, 350 patients were treated by MT and 125 patients received successful PCI procedures. Baseline clinical characteristics, including age, gender, hypertension, diabetes, dyslipidemia, familial history of CAD, prior MI, and CKD, were not significantly different in the two study groups. The usage of baseline medication was also similar. Compared to patients in the MT group, those who underwent successful procedures more often had two CTO lesions, CTO location in the left anterior descending artery, and low J-CTO score.

A total of 321 pairs of patients were matched among the patients with preserved LV systolic function after PSM. No considerable differences were found in the baseline clinical and lesion characteristics. Eighty-one pairs of patients were matched among the patients with LV systolic dysfunction. Similarly, the clinical baseline characteristics were not different between the successful PCI and medical therapy groups (Table 3).

**Table 3:** Baseline clinical, angiographic, and procedural characteristics of propensity-matched patients with and without left ventricular systolic dysfunction stratified according to management.

	**Propensity-matched patients with LVEF** **>45%**			**Propensity-matched patients with** **≤45%**		
	**Medical therapy**	**Successful PCI**	***P*-value**	**Medical therapy**	**Successful PCI**	***P*-value**
	**(*n* = 321)**	**(*n* = 321)**		**(*n* = 81)**	**(*n* = 81)**	
Age, years	64.0 ± 10.5	63.9 ± 10.0	0.820	64.2 ± 10.3	63.3 ± 10.7	0.608
Male	255 (79.4)	240 (74.8)	0.159	67 (82.7)	64 (79.0)	0.549
Smoking	138 (43.0)	131 (40.8)	0.576	40 (49.4)	49 (60.5)	0.155
Hypertension	226 (70.4)	218 (67.9)	0.494	51 (63.0)	51 (63.0)	1.000
Diabetes mellitus	113 (35.2)	91 (28.3)	0.062	33 (40.7)	35 (43.2)	0.750
Dyslipidemia	244 (76.0)	229 (71.3)	0.179	58 (71.6)	56 (69.1)	0.731
Familial history of CAD	39 (12.1)	43 (13.4)	0.636	7 (8.6)	7 (8.6)	1.000
Previous MI	77 (24.0)	88 (27.4)	0.320	37 (45.7)	38 (46.9)	0.875
CKD	17 (5.3)	17 (5.3)	1.000	9 (11.1)	11 (13.6)	0.633
Baseline medication						
Aspirin	311 (96.9)	309 (96.3)	0.664	79 (97.5)	78 (96.3)	0.650
Clopidogrel	302 (94.1)	308 (96.0)	0.277	70 (86.4)	77 (95.1)	0.058
Statin	306 (95.3)	304 (94.7)	0.717	80 (98.8)	80 (98.8)	1.000
β blocker	240 (74.8)	243 (75.7)	0.784	67 (82.7)	69 (85.2)	0.669
ACEI or ARB	209 (65.1)	205 (63.9)	0.741	57 (70.4)	56 (69.1)	0.864
One CTO lesion	290 (90.3)	281 (87.5)	0.314	63 (77.8)	64 (79.0)	0.849
Two CTO lesions	27 (8.4)	36 (11.2)	0.288	15 (18.5)	16 (19.8)	0.842
LAD	109 (34.0)	103 (32.1)	0.615	30 (37.0)	36 (44.4)	0.337
LCX	94 (29.3)	94 (29.3)	1.000	23 (28.4)	24 (29.6)	0.863
RCA	145 (45.2)	159 (49.2)	0.304	44 (54.3)	39 (48.1)	0.432
Multivessel disease	246 (76.6)	255 (79.4)	0.391	61 (75.3)	64 (79.0)	0.574
Proximal or mid CTO Location	220 (68.5)	222 (69.2)	0.865	62 (76.5)	61 (75.3)	0.854
Calcification	44 (13.7)	49 (15.3)	0.575	15 (18.5)	14 (17.3)	0.838
length ≥20 mm	182 (56.7)	199 (62.0)	0.172	56 (69.1)	52 (64.2)	0.505
J-CTO score	1.49 ± 1.18	1.49 ± 1.05	0.627	1.65 ± 1.17	1.52 ± 1.05	0.439
SYNTAX score	22.2 ± 8.9	21.4 ± 7.2	0.570	24.1 ± 8.6	23.3 ± 7.9	0.512

*Values are presented as the mean ± standard deviation or n (%)*.

*ACEI, angiotensin-converting enzyme inhibitor; ARB, angiotensin-receptor blocker; CAD, coronary artery disease; CKD, chronic kidney disease; CTO, chronic total occlusion; J-CTO, Japanese-chronic total occlusion; LAD, left ascending coronary artery; LCX, left circumflex coronary artery; LVEF, left ventricular ejection fraction; MI, myocardial infarction; PCI, percutaneous coronary intervention; RCA, right coronary artery*.

### Clinical Follow-Up

**Figure 1:**
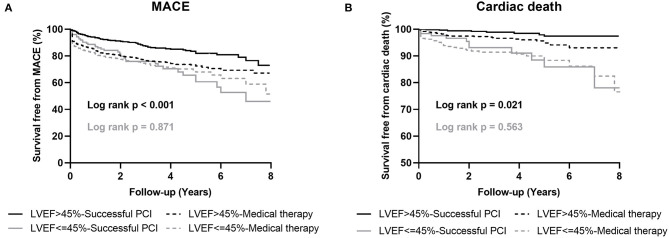
Kaplan-Meier curves for MACE **(A)** and cardiac death **(B)** during follow-up for successful CTO-PCI vs. medical therapy in patients with and without left ventricular systolic dysfunction. LVEF, left ventricular ejection fraction; MACE, major adverse cardiovascular events; PCI, percutaneous coronary intervention.

The median follow-up period was 2.6 years (interquartile range: 1.2–4.7 years). Among patients with preserved LV systolic function, the occurrence of MACE (successful CTO-PCI vs. MT: 14.2 vs. 23.9%, adjusted HR 0.63, 95% CI 0.48–0.83, *p* = 0.001) in the successful CTO-PCI group was significantly lower than that in the MT group. For cardiac mortality, the univariate analysis showed that patients in the MT group had a higher risk than the successful PCI patients, although multivariate analysis failed to demonstrate a significant difference (3.8 vs. 1.8%, adjusted HR 0.66, 95% CI 0.31–1.41, *p* = 0.286). For TVR, successful PCI was relatively inferior (adjusted HR 0.69, 95% CI: 0.50–0.96, *p* = 0.032) compared to MT. For MI, there was no significant difference between MT and successful PCI (adjusted HR 0.79, 95% CI: 0.50–1.23, *p* = 0.305; Table 4; Figure 1).

**Table 4:** Clinical outcomes of patients with LVEF >45% and with LVEF ≤45% stratified according to management.

**Patients with LVEF** **>45%**						
	**Medical therapy** ***n*** **=** **863**	**Successful PCI** ***n*** **=** **557**	**Crude HR (95% CI)**	***P*****-value**	Adjusted* **HR (95% CI)**	***P*****-value**
MACE	206 (23.9)	79 (14.2)	0.54 (0.41–0.70)	<0.001	0.63 (0.48–0.83)	0.001
Cardiac death	33 (3.8)	10 (1.8)	0.44 (0.21–0.90)	0.025	0.66 (0.31–1.41)	0.286
MI	70 (8.1)	32 (5.7)	0.69 (0.45–1.05)	0.088	0.79 (0.50–1.23)	0.305
TVR	131 (15.2)	56 (10.1)	0.61 (0.44–0.84)	0.002	0.69 (0.50–0.96)	0.032
**Patients with LVEF** **≤45%**						
	**Medical therapy** ***n*** **=** **350**	**Successful PCI** ***n*** **=** **125**	**Crude HR (95% CI)**	***P*****-value**	Adjusted** **HR (95% CI)**	***P*****-value**
MACE	101 (28.9)	37 (29.6)	0.96 (0.66–1.41)	0.874	1.05 (0.71–1.56)	0.792
Cardiac death	37 (10.6)	11 (8.8)	0.82 (0.41–1.61)	0.565	0.88 (0.43–1.83)	0.750
MI	31 (8.9)	14 (11.2)	2.06 (0.72–5.87)	0.176	2.21 (0.46–10.47)	0.317
TVR	46 (13.1)	21 (16.8)	1.23 (0.73–2.06)	0.432	1.25 (0.72–2.16)	0.412

*Values are given as numbers and percentages*.

Adjusted* by age, diabetes mellitus, previous MI, taking clopidogrel, CTO in LAD, multivessel disease, proximal or mid CTO, calcification, J-CTO score and SYNTAX score.

Adjusted** by CKD, number of CTO lesion, CTO in LAD, multivessel disease, calcification, J-CTO score and SYNTAX score.

*CI, confidence interval(s); CKD, chronic kidney disease; CTO, chronic total occlusion; HR, hazard ratio; J-CTO, Japanese-chronic total occlusion; LAD, left ascending coronary artery; MACE, major adverse cardiovascular events; MI, myocardial infarction; LVEF, left ventricular ejection fraction; PCI, percutaneous coronary intervention; TVR, target-vessel revascularization*.

Among the patients with LV systolic dysfunction, MACE occurred in 101 (28.9%) patients with MT and 37 (29.6%) patients with successful PCI. In both univariate and multivariate analyses, no differences were observed in MACE (adjusted HR 1.05, 95% CI: 0.71–1.56, *p* = 0.792), cardiac mortality (adjusted HR 0.88, 95% CI: 0.43–1.83, *p* = 0.750), MI (adjusted HR 2.21, 95% CI: 0.46–10.47, *p* = 0.317), and TVR (adjusted HR 1.25, 95% CI: 0.72–2.16, *p* = 0.412; Table 4; Figure 1).

**Figure 2:**
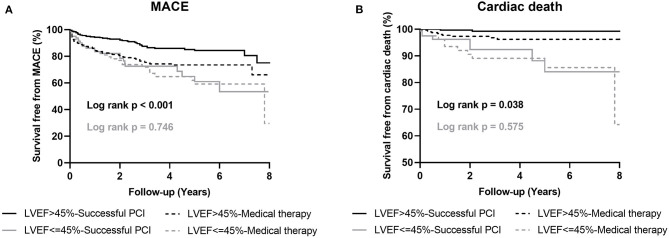
Kaplan-Meier curves for MACE **(A)** and cardiac death **(B)** during follow-up for successful CTO-PCI vs. medical therapy in propensity-matched patients with and without left ventricular systolic dysfunction. LVEF, left ventricular ejection fraction; MACE, major adverse cardiovascular events; PCI, percutaneous coronary intervention.

Among patients with preserved LV systolic function after PSM, those who received MT exhibited a higher rate of MACEs (HR 0.48, 95% CI 0.33–0.71, *p* < 0.001) and cardiac death (HR 0.28, 95% CI 0.07–1.01, *p* = 0.034) compared to patients in the successful CTO-PCI group. Among the patients with LV systolic dysfunction, the incidence of MACE (HR 0.91, 95% CI 0.52–1.59, *p* = 0.747) and cardiac death (HR 0.76, 95% CI 0.29–2.00, *p* = 0.575) was similar between the two groups (Table 5; Figure 2).

**Table 5:** Clinical outcomes of propensity-matched patients with and without left ventricular systolic dysfunction stratified according to management.

**Patients with LVEF** **>45%**				
	**Medical therapy** ***n*** **=** **321**	**Successful PCI** ***n*** **=** **321**	**HR (95% CI)**	***P*****-value**
MACE	77 (24.0)	42 (13.1)	0.48 (0.33–0.71)	<0.001
Cardiac death	10 (3.1)	3 (0.9)	0.28 (0.07–1.01)	0.034
MI	37 (11.5)	19 (5.9)	0.47 (0.21–0.82)	0.006
TVR	49 (15.3)	30 (9.3)	0.55 (0.35–0.87)	0.010
**Patients with LVEF** **≤45%**				
	**Medical therapy** ***n*** **=** **81**	**Successful PCI** ***n*** **=** **81**	**HR (95% CI)**	***P*****-value**
MACE	27 (33.3)	24 (29.6)	0.91 (0.52–1.59)	0.747
Cardiac death	10 (12.3)	7 (8.6)	0.76 (0.29–2.00)	0.575
MI	11 (13.6)	10 (12.3)	0.95 (0.40–2.25)	0.916
TVR	10 (12.3)	13 (16.0)	1.33 (0.58–3.06)	0.498

*Values are presented as n (%)*.

*CI, confidence interval(s); HR, hazard ratio; MACE, major adverse cardiovascular events; MI, myocardial infarction; LVEF, left ventricular ejection fraction; PCI, percutaneous coronary intervention; TVR, target-vessel revascularization*.

The outcomes in patients with LVEF <40, 40–50, and >50% are reported in Table 6. There were 430 (22.7%) patients with mid-range LVEF (40–50%). There were no significant differences between MT and successful PCI in patients with mid-range LVEF in MACE (adjusted HR 0.79, 95% CI: 0.51–1.22, *p* = 0.298) and cardiac death (adjusted HR 0.67, 95% CI: 0.23–1.90, *p* = 0.458) incidence (Table 6). Similarly, no difference was observed in MACE incidence (adjusted HR 0.89, 95% CI: 0.55–1.44, *p* = 0.648) in patients with LVEF <40%. However, among patients with LVEF ≥50%, the MACE rate (MT vs. successful CTO-PCI: 22.7 vs. 13.4%, adjusted HR 0.58, 95% CI 0.43–0.78, *p* < 0.001) in the successful CTO-PCI group was significantly lower than that in the MT group.

**Table 6:** Clinical outcomes of patients with different LVEF stratified according to management.

**Patients with LVEF** **<40%**				
	**Medical therapy** ***n*** **=** **242**	**Successful PCI** ***n*** **=** **80**	**HR (95% CI)**	***P*****-value**
MACE	74 (30.5)	24 (30.0)	0.89 (0.55–1.44)	0.648
Cardiac death	27 (11.1)	8 (10.8)	0.95 (0.40–2.25)	0.916
MI	24 (9.9)	10 (12.5)	1.25 (0.58–2.70)	0.558
TVR	31 (12.8)	14 (17.5)	1.16 (0.59–2.25)	0.661
**Patients with 40** **≤** **LVEF** **<50%**				
	**Medical therapy** ***n*** **=** **283**	**Successful PCI** ***n*** **=** **147**	**HR (95% CI)**	***P*****-value**
MACE	76 (26.9)	31 (21.1)	0.79 (0.51–1.22)	0.298
Cardiac death	17 (6.0)	3 (0.9)	0.67 (0.23–1.90)	0.458
MI	26 (9.2)	11 (7.5)	0.80 (0.37–1.70)	0.562
TVR	44 (15.5)	21 (14.3)	0.88 (0.51–1.53)	0.672
**Patients with LVEF** **≥50%**				
	**Medical therapy** ***n*** **=** **775**	**Successful PCI** ***n*** **=** **498**	**HR (95% CI)**	***P*****-value**
MACE	176 (22.7)	67 (13.4)	0.58 (0.43–0.78)	<0.001
Cardiac death	28 (3.6)	8 (1.6)	0.56 (0.24–1.30)	0.181
MI	60 (7.7)	29 (5.8)	0.91 (0.56–1.45)	0.694
TVR	113 (14.5)	46 (9.2)	0.60 (0.42–0.86)	0.005

*Values are presented as n (%)*.

*CI, confidence interval(s); HR, hazard ratio; MACE, major adverse cardiovascular events; MI, myocardial infarction; LVEF, left ventricular ejection fraction; PCI, percutaneous coronary intervention; TVR, target-vessel revascularization*.

Analysis of MT compared to initial CTO-PCI (including successful CTO PCI and failed CTO PCI) in CTO patients with and without LV systolic dysfunction was also performed (Table 7). Multivariable Cox regression analysis showed that when compared to MT, initial CTO-PCI was associated with fewer MACEs in both patients with preserved (adjusted HR 0.60, 95% CI 0.48–0.75, *p* < 0.001) or reduced (adjusted HR 0.65, 95% CI 0.46–0.93, *p* = 0.021) LV systolic function.

**Table 7:** Clinical outcomes of patients with and without left ventricular systolic dysfunction stratified according to medical therapy or initial CTO-PCI.

**Patients with LVEF** **>45%**				
	**Medical therapy** ***n*** **=** **863**	**CTO-PCI** ***n*** **=** **917**	**HR (95% CI)**	***P*****-value**
MACE	206 (23.9)	136 (14.8)	0.60 (0.48-0.75)	<0.001
Cardiac death	33 (3.8)	20 (2.2)	0.81 (0.45-1.47)	0.504
MI	70 (8.1)	59 (6.4)	0.95 (0.66-1.36)	0.798
TVR	131 (15.2)	85 (9.3)	0.56 (0.43-0.75)	<0.001
**Patients with LVEF** **≤45%**				
	**Medical therapy** ***n*** **=** **350**	**CTO-PCI** ***n*** **=** **241**	**HR (95% CI)**	***P*****-value**
MACE	101 (28.9)	49 (20.3)	0.65 (0.46-0.93)	0.021
Cardiac death	37 (10.6)	20 (8.3)	0.81 (0.45-1.45)	0.486
MI	31 (8.9)	17 (7.1)	0.82 (0.45-1.51)	0.534
TVR	46 (13.1)	24 (10.0)	0.68 (0.41-1.14)	0.152

*Values are presented as n (%)*.

*CI, confidence interval(s); CTO, chronic total occlusion; HR, hazard ratio; MACE, major adverse cardiovascular events; MI, myocardial infarction; LVEF, left ventricular ejection fraction; PCI, percutaneous coronary intervention; TVR, target-vessel revascularization*.

**Figure 3:**
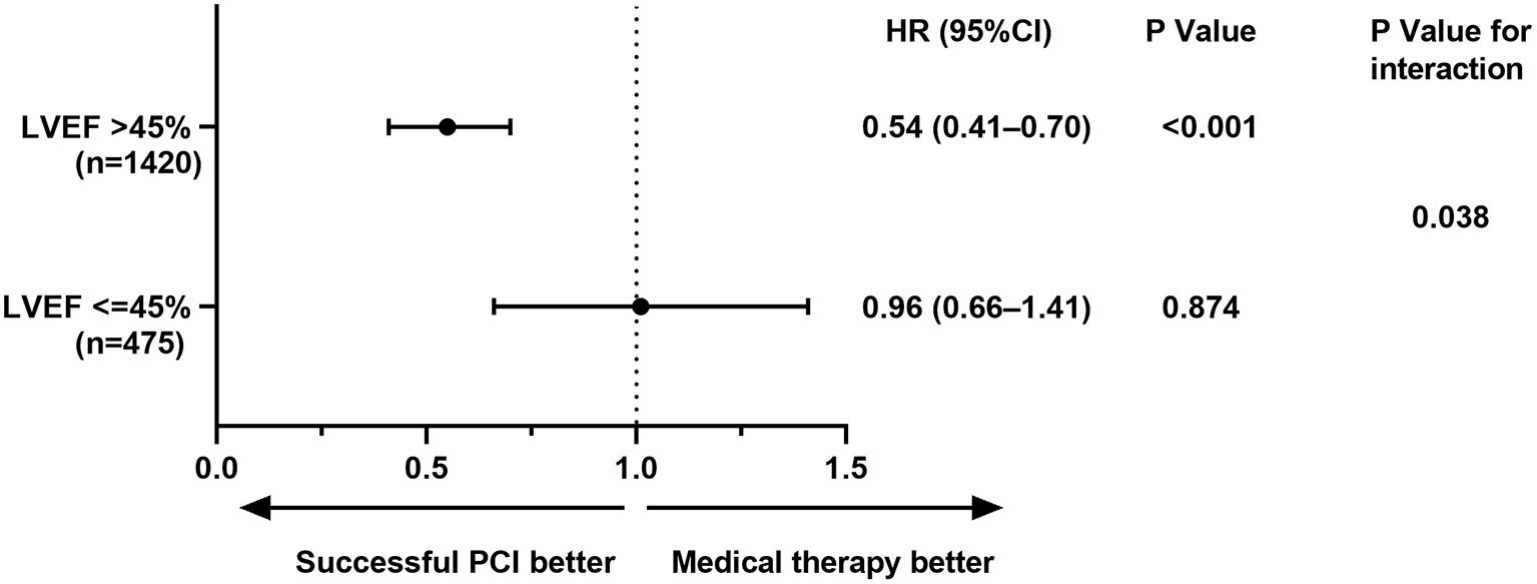
Left ventricular systolic function subgroup analysis for MACE. CI, confidence interval(s); HR, hazard ratio; LVEF, left ventricular ejection fraction; MACE, major adverse cardiovascular events; PCI, percutaneous coronary intervention.

A significant interaction was observed between LV function and therapeutic strategy for MACE (*p* = 0.038). Survival free from MACEs in successful CTO-PCI patients was significant among patients with preserved LV systolic function, but not in patients with LV systolic dysfunction (Figure 3).

## Discussion

The principal clinical findings in this large cohort study can be summarized as follows: (1) CTO patients with LV systolic dysfunction had more comorbidities and complex lesions compared to patients with preserved LV systolic function; (2) successful CTO PCI reduced MACE occurrence compared to MT alone in patients with preserved LV systolic function; and (3) successful CTO recanalization was not associated with reduced MACE incidence or cardiovascular mortality compared to MT alone in CTO patients with LV systolic dysfunction.

In terms of the baseline demographic characteristics in the present study, patients with LV systolic dysfunction had more comorbidities, such as previous MI and CKD, and presented with more complex lesions, including multivessel coronary artery disease and long occlusion. This was consistent with the findings from Tajstra et al. and Toma et al., which revealed that CTO patients with low LVEF had a higher prevalence of previous MI, CKD, multivessel disease, and severely calcified lesions (19, 20). Of note, these patients are more likely to encounter contrast-induced nephropathy (CIN). Liu et al. performed preprocedural risk scoring to predict CIN after CTO intervention based on three factors, and LV dysfunction was included (21). These features were related to decreased success rates and increased periprocedural complications from CTO PCI (22), which have been considered by cardiac interventionist, and they were more often reluctant to perform more complex CTO procedures in high-risk subjects compared to those with preserved LVEF. Indeed, the rate of CTO PCI was only 3.5% in the COMMIT-HF registry, which enrolled 675 patients with systolic heart failure (20). In addition, the use of β-blocker and ACEI or ARB was more common among patients with LV dysfunction. A recent random trial and meta-analysis demonstrated that these medications can improve LVEF and reduce adverse outcomes by reducing heart rate, altering vascular function, modifying neuro-endocrine response to heart failure, and reversing myocardial remodeling (23, 24).

CAD with severe LV dysfunction is associated with high morbidity and adverse outcomes, increased risk of ventricular arrhythmias, cardiogenic death, decreased quality of life, and high medical costs (20). As a subset of CAD, the presence of coronary CTO in patients with LV systolic dysfunction was related to a significant increase in all-cause death and cardiovascular mortality (20). In patients with CTO undergoing PCI, those with LV dysfunction encountered higher MACE occurrence and 3-fold risk of cardiac mortality (10.1 vs. 3.0%, *p* < 0.001). Recent registry studies also underscored that reduced LV systolic function is an independent predictor for mortality among patients undergoing CTO PCI (4, 25).

Improvement in LVEF after CTO recanalization in patients with LV dysfunction is still controversial. Recently, a weighted meta-analysis was performed by merging 34 studies and including a total of 2,243 patients to address the impact of CTO revascularization. It revealed that the absolute LVEF points increased by 4.44% after successful CTO recanalization (26). This was consistent with the findings in the latest meta-analysis performed by Megaly et al. in 2018 (5). Nevertheless, the EXPLORE trial enrolled 304 patients with acute ST-segment elevation MI who underwent primary PCI and had concurrent CTO (27). Patients were randomized and assigned to early CTO-PCI or conservative treatment within 7 days from the infarction groups. After a follow-up of 4 months, there was no statistically significant difference in mean LVEF between the two groups. More recently, CTO patients in the REVASC trial were randomly assigned to revascularization or no revascularization of CTO groups. No benefit for CTO revascularization was observed with regard to segmental wall thickening or regional and global LV function after 6 months (28).

The choice of optimal treatment strategy for CTO patients with LV dysfunction is often challenging. Of note, current guidelines for myocardial revascularization do not provide any evidence-based recommendation in terms of the most appropriate treatment strategy in such high-risk patients (29, 30). A recent study based on 436 CTO patients with reduced LVEF showed that there were differences in clinical outcomes, such as death, MI, and stroke, between revascularized and not revascularized CTO (31). On the other hand, Galassi et al. performed a study including 839 patients undergoing CTO PCI attempts and found that successful CTO recanalization was not associated with improved midterm clinical outcome, which included cardiac death, non-fatal MI, TVR, stroke, and MACCEs in patients with LVEF of 35–50% (32). In addition, Lee et al. and Yamamoto et al. have also reported that prevalence of death or MACEs was not different in patients undergoing successful CTO-PCI compared to failed procedures or MT (33, 34). Even in the DECISION-CTO and the Euro-CTO trials, which compared revascularization to optimal MT for the treatment of CTO, the incidence of MACE, death, and repeated revascularization was comparable between the two groups (35, 36).

The number of CTO patients with LV systolic dysfunction is significant, and large contemporary CTO registries have reported that 40–53% of patients had LV systolic dysfunction (9, 12). However, a comparison of successful CTO PCI vs. optimal MT effects on long-term clinical outcomes in patients with LV systolic dysfunction has not been performed until now, and the optimal treatment strategy for these high-risk subjects is unknown. In the present study, a good long-term outcome was achieved among patients with preserved LV function, but not with LV dysfunction, when they were referred for revascularization. These results were verified by both multivariable Cox regression analysis and PSM. Therefore, these findings are more convincing when compared to previous studies. Subgroup analysis showed that there was a significant interaction between LV function and therapeutic strategy for MACE, suggesting that superiority of revascularization over conservative therapy may be dependent on LV function. Notably, a failed CTO procedure is known to be related to a higher incidence of procedural complications and adverse events, leading to poor prognosis, especially in patients with high-risk factors (37, 38). Therefore, in our study, patients with failed CTO-PCI were excluded. Clinical outcomes between successful CTO PCI and MT (CTO-PCI not attempted) groups were investigated to better reflect the overall risk of patients with coronary CTO. Our study differs from previous studies (19, 31) and is more reflective of the “real world” of clinical practice. To the best of our knowledge, this is the first large cohort study reporting on the long-term outcomes of successful recanalization compared to MT in unselected CTO patients with and without LV dysfunction.

Galassi et al. confirmed that patients with LV dysfunction had a higher tamponade-induced coronary perforation (2.3 vs. 1.4%) and non-Q-wave MI (1.9 vs. 0.5%) incidence compared to those with preserved LV systolic function when they were undergoing CTO-PCI (32). These results indicate that CTO-PCI has to be carefully considered, taking into account multiple comorbidities, complex coronary lesions, operative complications, and clinical outcomes in this high-risk patient population.

We noticed that the frequency of MACE is actually lower in failed CTO-PCI cases (12/116: 10.3%) than in successful CTO-PCI cases (37/125: 29.6%) with reduced LV systolic function. As we can see the Kaplan-Meier survival curve of MACE in Figure 1 and outcomes in successful recanalization group in Table 4, we found that TVR and MI were the predominant determinant of MACE. In our study, rate of multivessel disease was 80%, which was associated with high prevalence of MI, and some patients underwent CABG during follow-up. In addition, 70.9% patients were with proximal or mid CTO lesions, and more than half of them were with mid CTO lesions. The left (29.1%) patients were with distant CTO lesions. TVR was defined as repeat revascularization in the target vessel and included any emergency or elective CABG or repeat PCI according to Standard Definitions. Many patients were with prior coronary stenosis, new lesion or lesion progression before CTO location and they received following CABG or repeat PCI for these stenotic lesions when they had new or persistent angina, or even recent repeated MI after failed PCI and optimal medical therapy. Stent thrombosis and in-stent restenosis, which caused by what stent undersizing, presence of residual dissection, impaired TIMI flow and residual disease proximal or distal to the stent lesion, were main reasons for TVR, and often appeared in the first few months (39). The rate of TVR observed in our study was consistent with previously reported data, and TVR was the predominant determinant of MACE (19, 40, 41). In a recent study, Pinto et al. reported that, in patients with LV systolic dysfunction, the TVR rate was 29.8% in revascularized CTO group and 15.5% in not revascularized CTO group (31). In addition, Galassi et al. also reported, in CTO patients with low LVEF, the rate of TVR was 6.1% in successful CTO PCI group but 0% in failed CTO PCI group (32), which was consistent with our study.

Some limitations of the study merit consideration. First, its non-randomized nature may cause a selection bias, which may influence the results due to confounding factors, though the multivariable Cox regression analysis and PSM were performed. Second, the percentage of patients with preserved LVEF experiencing diastolic dysfunction was not reported, and it may influence clinical outcomes. Third, the data on LVEF improvement and reverse remodeling during follow-up in patients with and without LV systolic dysfunction were not reported in the study. Fourth, since the study's time period was long (12 years), the changes in PCI materials and techniques or MT (such as anti-thrombotic medication or heart failure medication) may have an impact on clinical outcomes, though we have considered these factors in our previous studies (7, 18).

## Conclusions

Compared to MT alone in patients with native coronary CTOs, successful CTO-PCI may reduce the risk of MACE in patients with preserved LV systolic function, but not in patients with LV dysfunction. Well-designed and powered randomized investigations are needed to verify these findings from our large registry and further define the management strategy in this high-risk population.

## Data Availability Statement

The raw data supporting the conclusions of this article will be made available by the authors, without undue reservation.

## Ethics Statement

The studies involving human participants were reviewed and approved by First Affiliated Hospital of Dalian Medical University. The patients/participants provided their written informed consent to participate in this study.

## Author Contributions

LG, SM, HL, and RH prepared the manuscript. All authors contributed to the data collection, analyses, edited the draft manuscript, and approved the final manuscript.

## Conflict of Interest

The authors declare that the research was conducted in the absence of any commercial or financial relationships that could be construed as a potential conflict of interest.
